# *Bartonella quintana* detection among arthropods and their hosts: a systematic review and meta-analysis

**DOI:** 10.1186/s13071-024-06413-3

**Published:** 2024-08-02

**Authors:** Carl Boodman, Nitin Gupta, Johan van Griensven, Wim Van Bortel

**Affiliations:** 1 University of Manitoba, Winnipeg, MB Canada; 2 Institute of Tropical Medicine, Antwerp, Belgium; 3 University of Antwerp, Antwerp, Belgium; 4 Department of Infectious Disease, Kasturba Medical College, Manipal Academy of Higher Education, Manipal, India

**Keywords:** Arthropods, Vector, *Bartonella quintana*, Trench fever, Lice

## Abstract

**Background:**

*Bartonella quintana* is a body louse-borne bacterium causing bacteremia and infective endocarditis. We aimed to describe *B. quintana* detection among arthropods and their hosts.

**Methods:**

We searched databases in PubMed Central/MEDLINE, Scopus, Embase, and Web of Science from January 1, 1915 (the year of *B. quintana* discovery) to January 1, 2024, to identify publications containing specific search terms relating to *B. quintana* detection among arthropods. Descriptive statistics and meta-analysis of pooled prevalence using random-effects models were performed for all arthropods and body and head lice.

**Results:**

Of 1265 records, 62 articles were included, describing 8839 body lice, 4962 head lice, and 1692 other arthropods, such as different species of fleas, bedbugs, mites, and ticks. Arthropods were collected from 37 countries, of which 28 had arthropods with *B. quintana* DNA. Among articles that reported *B. quintana* detection among individual arthropods, 1445 of 14,088 (0.1026, 95% CI [0.0976; 0.1077]) arthropods tested positive for *B. quintana* DNA, generating a random-effects model global prevalence of 0.0666 (95% CI [0.0426; 0.1026]). Fifty-six studies tested 8839 body lice, of which 1679 had *B. quintana* DNA (0.1899, 95% CI [0.1818; 0.1983]), generating a random-effects model pooled prevalence of 0.2312 (95% CI [0.1784; 0.2843]). Forty-two studies tested 4962 head lice, of which 390 head lice from 20 studies originating from 11 different countries had *B. quintana* DNA (0.0786, 95% CI [0.0713; 0.0864]). Eight studies detected *B. quintana* DNA exclusively on head lice. Five studies reported greater *B. quintana* detection on head lice than body lice; all originated from low-resource environments.

**Conclusions:**

*Bartonella quintana* is a vector-borne bacterium with a global distribution, disproportionately affecting marginalized populations. *Bartonella quintana* DNA has been detected in many different arthropod species, though not all of these arthropods meet criteria to be considered vectors for *B. quintana* transmission. Body lice have long been known to transmit *B. quintana*. A limited number of studies suggest that head lice may also act as possible vectors for *B. quintana* in specific low-resource contexts.

**Graphical Abstract:**

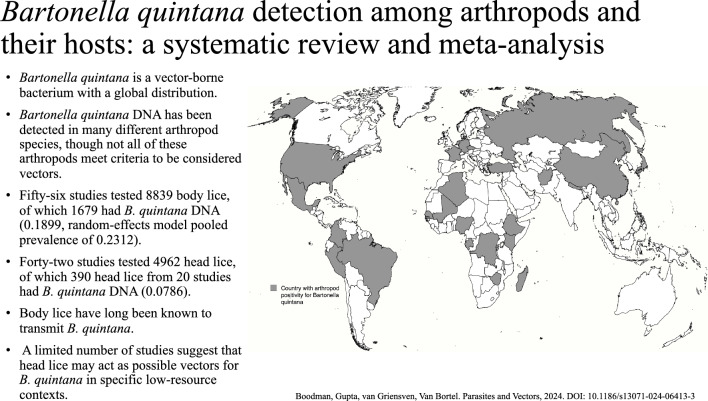

**Supplementary Information:**

The online version contains supplementary material available at 10.1186/s13071-024-06413-3.

## Background

*Bartonella quintana* is a vector-borne, intracellular, Gram-negative bacillus [1]. The bacterium has a tropism for erythrocytes and endothelial cells, causing clinical disease in the form of infective endocarditis, bacillary angiomatosis, and chronic bacteremia [2–5]. Because of *B. quintana*’s intracellular localization and sluggish replication, the pathogen is not identified by blood culture with routine 5-day incubation [6]. *Bartonella quintana* is thus described as a major cause of culture-negative endocarditis and predominantly requires molecular techniques for species-level identification [6, 7].

*Bartonella quintana* was discovered in 1915 as the cause of trench fever, a relapsing febrile illness afflicting 1 million soldiers during World War I (WWI) [8, 9]. Soon after the bacterium’s detection, the War Office Committee on Trench Fever determined in 1917 that *B. quintana* was transmitted via body lice: “A small quantity of the excreta of infected lice rubbed into scratches will almost invariably reproduce the disease in a healthy man” [10]. Experimental infections of laboratory-raised body lice fed with blood from patients with trench fever produced “rickettsial bodies” in the lice [10]. As described in these early experiments, transmission of *B. quintana* entails the inoculation of *B. quintana*-infected body louse feces into skin abrasions and mucous membranes [1, 2]. Once in the human host, the bacillus infects erythrocytes, causing chronic bacteremia [3, 11]. The longest documented duration of *B. quintana* bacteremia is 8 years, but most recent studies describe periods of up to 1 year [3, 11]. As *B. quintana* predominantly affects individuals with body louse infestation (pediculosis corporis), the bacterial infection is associated with poverty, overcrowding, and barriers to maintaining personal hygiene such as insufficient access to running water [12–14]. Contemporary outbreaks of *B. quintana* infection have occurred among populations experiencing homelessness, refugees in low-income countries, and Canadian Indigenous communities with limited access to adequate housing and running water [1, 13].

For over 100 years, dogma has maintained that the human body louse (*Pediculus humanus humanus)* is the arthropod vector for *B. quintana* despite increasing reports of *B. quintana* DNA detection from human head lice (*P. humanus capitis*) and other arthropods, including cat fleas, pigeon mites, bedbugs, and ticks of various species [2, 15–20]. While head and body lice belong to the same species (*P. humanus*) and are morphologically identical, they belong to two separate ecotypes, inhabiting different ecological niches [21]. Head lice live on head hair, and body lice live in clothing seams [21]. Some experts refer to the latter more accurately as clothing lice [21]. Both ecotypes of lice feed intermittently on human blood [21]. Beyond *B. quintana*, body lice transmit epidemic typhus (*Rickettsia prowazekii*) and louse-borne relapsing fever (*Borrelia recurrentis*) [1]. Head lice are not known to transmit pathogens and are thus not viewed as a major health hazard [21, 22].

For non-*Pediculus* arthropods, individual studies of macaque lice, bedbugs, pigeon mites, ticks, and fleas have detected *B. quintana* DNA using molecular methods, but no studies have exhaustively documented the detection of *B. quintana* among different arthropods and analyzed the results according to arthropod species, region, and host characteristics [15, 19, 23, 24].

This systematic review aimed to describe *B. quintana* detection among different arthropod species and their hosts.

## Methods

### Systematic literature search strategy

We searched databases in PubMed Central/MEDLINE, Scopus, Embase, and Web of Science from January 1, 1915 (the year of *B. quintana* discovery) to January 1, 2024, to identify publications containing specific search terms relating to *B. quintana* detection among arthropods. We searched for titles and abstracts using the following search string, with associated Medical Subject Headings (MeSH) terms and Boolean operators for each database: {(*Bartonella quintana* OR *Rochalimaea quintana* OR *Rickettsia quintana* OR Trench fever) AND (Arthropod OR Insect OR Vector OR Ectoparasite OR Lice OR Flea OR Mite OR Fly OR Bedbug OR Tick)}. Moreover, we searched reference lists of selected publications to identify other articles. No language restrictions were placed, though search terms were run in English. This review followed the Preferred Reporting Items for Systematic Reviews and Meta-Analyses (PRISMA) guidelines for systematic literature reviews and was registered in the International Prospective Register of Systematic Reviews (PROSPERO; identifier CRD42024503951) [25, 26].

### Study selection

Laboratory confirmation of *B. quintana* at the species level was required for inclusion (Additional file 1). Studies that tested arthropods for *B. quintana* but lacked arthropod identification at the genus level were excluded. In vitro studies where arthropods were raised in a laboratory (not collected in the environment) were excluded, as were review articles describing previously published data. There was no spatial limitation.

### Article review

Article titles and abstracts were screened by two individuals (CB, NG) to determine eligibility for full-text review. Full texts of the articles included after title/abstract screening were reviewed by two independent reviewers (CB, NG). Reviewer discrepancies were resolved mutually and by discussion with a third reviewer.

### Quality assessment for included studies

Two reviewers (CB, NG) assessed articles for quality using the modified JBI critical appraisal checklist for methodological quality and potential bias (Additional file 1) [27]. Studies that failed to meet modified JBI criteria were excluded from the primary analysis.

### Data extraction

Data were manually extracted from the included articles by one author (CB) using Microsoft Excel (2019, version 16.72) and corroborated by a second author (NG) who signaled inconsistencies, which were resolved through discussion. For each included reference, we extracted the following:

- Data relating to the publication: last name of study’s first author, year of publication, country, and continent where data were acquired.
- Data relating to arthropod analysis: arthropod genus and species (and louse ecotype and clade), method of arthropod identification, co-infestation of body lice with head lice (for studies that analyzed both body and head lice), total number of arthropods tested, number of body and head lice, and number of arthropod pools (for studies that pooled arthropods).
- Data relating to *B. quintana* detection: number and percentage of arthropods with *B. quintana* detection, method of *B. quintana* identification, and associated molecular targets.
- Data relating to co-pathogens: results of testing of other pathogens from arthropod samples.
- Data relating to host: host species, number of hosts, host age category, and key population (e.g., homelessness, for human studies), number and percentage of hosts with *B. quintana*-infected arthropods, host bacteremia/presence of *B. quintana* DNA in host blood, and evidence of host clinical disease through symptom reporting.

Duplicate entries were prevented by consolidating identical data summarized in different articles into one record, prioritizing the first publication. Studies that reported data from multiple countries were divided by country to facilitate geographical analysis. Whenever applicable, data analyses were separated by studies that tested individual arthropods versus those that tested arthropod pools.

### Statistical analysis

Descriptive statistics and meta-analysis of prevalence using random-effects models were performed post hoc using R version 4.2.2 software (2022-10-31). The random-effects model included an inverse variance method with a restricted maximum-likelihood estimator, logit transformation, a normal approximation confidence interval (CI) for individual studies, and a continuity correction of 0.5 for studies with zero cell frequencies. The global and continent-specific pooled prevalence of *B. quintana* detection among arthropods was performed as well as the pooled prevalence for body and head lice. Heterogeneity among eligible articles was performed using Cochran’s Q statistic (*P*-value < 0.10 for statistical significance) and *I*^2^ index. The Chi-square test with Yates correction was used to analyze categorical variables, specifically to compare *B. quintana* detection between head and body lice (*P* ≤ 0.05 considered statistically significant).

## Results

### Search results

**Fig. 1:**
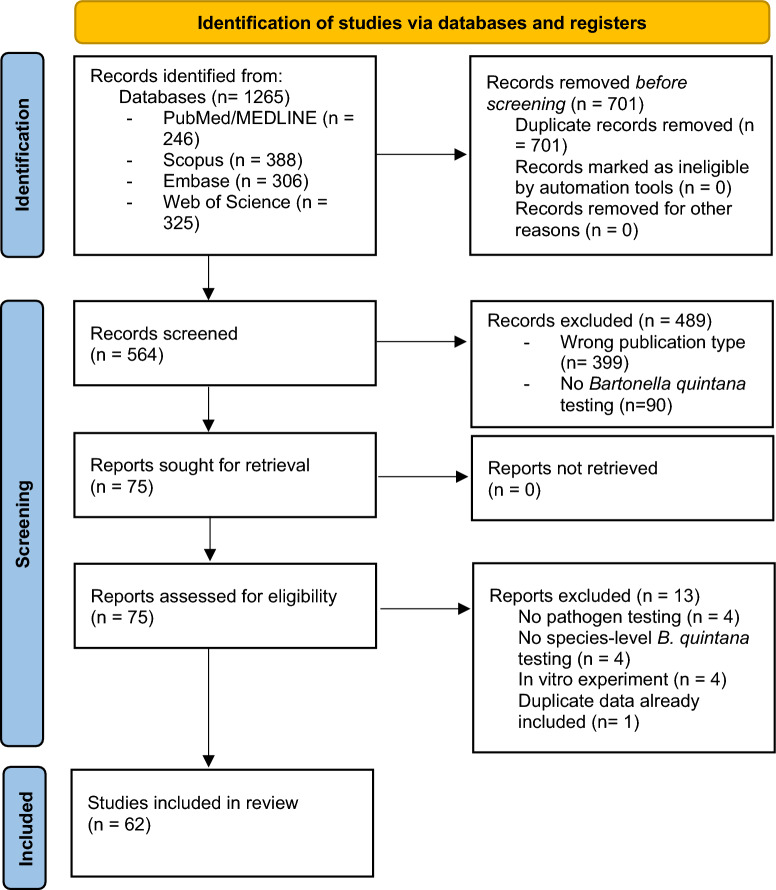
PRISMA 2020 flow diagram for this new systematic review which included searches of databases and registers

We identified 1265 articles through the database search (Fig. 1). After 701 duplicate articles were removed, 564 articles remained for title and abstract screening. After review of titles/abstracts and full texts, 62 publications met inclusion criteria, describing 15,493 arthropods tested for *B. quintana*. Fifty-four articles reported data on 14,088 individual arthropods, and eight reported data on arthropod pools, adding 1405 arthropods [17, 28]. The included articles were published between 1961 and 2023. In some cases, multiple different studies reported data from arthropods collected from the same country [29, 30]. Twenty-one studies tested multiple types of arthropods (e.g., body and head lice, body lice, and non-louse arthropods).

### Host species and characteristics

This review included 8735 hosts, of which 7673 (0.8784, 95% CI [0.8714; 0.8852]) were human and 1062 were non-human. All but eight studies tested arthropods from human hosts. Nineteen studies included children: 12 studies tested arthropods from adults and children, and seven studies exclusively tested children. Seventeen studies describe human hosts as having experienced homelessness [17]. Five studies describe participants as being refugees [31, 32]. Two studies describe hosts as being incarcerated and two as coming from a rural Indigenous community [33]. Regarding non-human hosts, two studies tested arthropods from cats, two from non-human primates (one from rhesus macaques and one from *Cercopithecus cephus* monkeys), two from rodents, one from boars, and one from birds. Two hundred ninety-three hosts were infested with arthropods with evidence of *B. quintana* infection, of which all but four hosts were human.

### Arthropod and pathogen identification

Arthropods were molecularly identified using polymerase chain reaction (PCR) in 46 of 62 studies (74.19%). Matrix-assisted laser desorption/ionization time-of-flight mass spectrometry was used in one study, and the remaining 15 studies identified arthropods morphologically using taxonomic keys. Twenty-seven studies ascertained louse clade using molecular analysis of the mitochondrial cytochrome b (*cytB*) gene. Clade A was identified in all but three studies, and 10 studies identified clade A in addition to at least one other.

PCR was used to determine the presence of pathogens, including *B. quintana*, in all but one study. The most common PCR targets for *B. quintana* were the internal transcribed spacer gene (ITS, often used as an initial screening for *Bartonella* genus), the putative targeted effector protein gene (*yopP*), the citrate synthase gene (*gltA*), and the 3-oxoacyl-[acyl-carrier-protein] synthase gene (*fabB*). Thirty-seven studies used multiple molecular targets to confirm *B. quintana*. Twenty-four studies combined ITS for *Bartonella* genus with a second target for *B. quintana* species. *Bartonella quintana* was identified using culture from arthropod samples in one French study that identified bacterial colonies with 16S ribosomal RNA (rRNA) sequencing [34].

### Geographical distribution

**Fig. 2:**
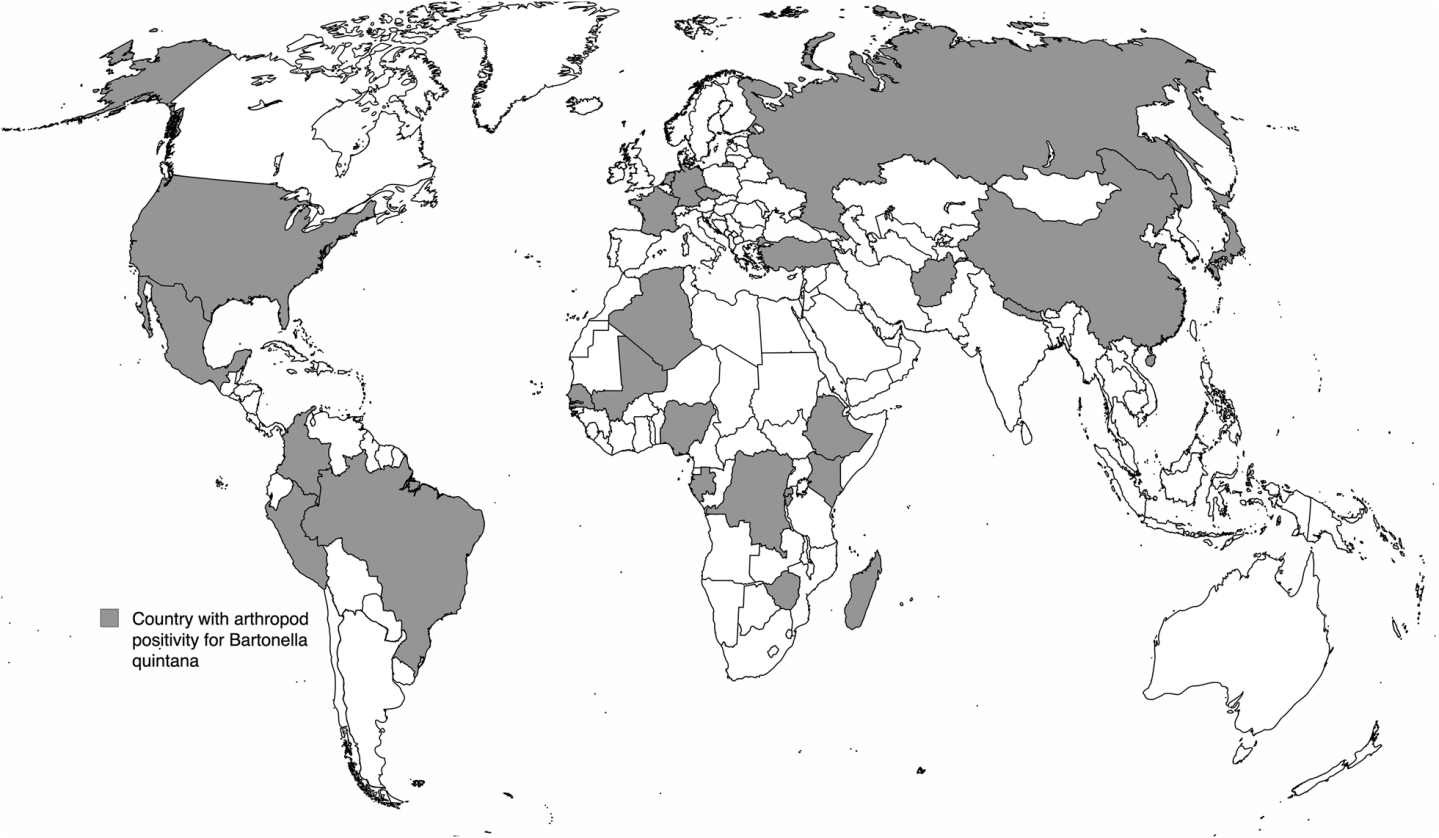
Map of countries reporting arthropods testing positive for *B. quintana*. Interactive map with data linked to original publication (author, year), arthropod species/ecotype, and positivity for *B. quintana* is available here: https://www.google.com/maps/d/u/0/edit?mid=1NRbIN72IDnbgxjdytjWVycd7_LmJx9k&usp=sharing

Arthropods were collected from 37 countries, of which 28 had arthropods with evidence of *B. quintana* infection (Fig. 2). Arthropods with *B. quintana* were reported from every continent except Oceania and Antarctica (Table 1). Arthropods from nine countries (Australia, South Korea, Thailand, Malaysia, Italy, Tanzania, Guinea, St. Kitts, Corsica^1^) were tested for *B. quintana* without reported detection (Additional file 2). Among articles that reported individual arthropods, *B. quintana* was detected among 1445 of 14,088 (0.1026, 95% CI [0.0976; 0.1077]) arthropods, generating a random-effects model global prevalence of 0.0666 (95% CI [0.0426; 0.1026]). High heterogeneity was found among studies (*I*^2^ = 93.3% [92.1%; 94.3%], Cochran’s Q, *P* < 0.0001) (Additional file 3) [35]. Africa had the most country-specific reports, with 43 reports describing 7040 arthropods. Of these, 703 (0.1000, 95% CI [0.0929; 0.1071]) tested positive for the presence of *B. quintana* DNA, representing a random-effects model prevalence of 0.0615 (95% CI [0.0311; 0.1180]), with elevated heterogeneity (*I*^2^ = 94.6% [93.5%; 95.5%], Cochran’s *Q*, *P* < 0.0001). The continent with the fewest publications was Oceania, which had only two reports describing five arthropods and no arthropods with *B. quintana* DNA.

**Table 1:** Global and regional arthropod prevalence for *Bartonella quintana*

Continent	No. reports	No. arthropods	No. Bq+	Bq proportion [95% CI]	Random-effects model proportion [95% CI]
Africa	43	7040	703	0.1000 [0.0929; 0.1071]	0.0615 [0.0311; 0.1180]
Europe	17	4062	321	0.0790 [0.0709; 0.0877]	0.0522 [0.0218; 0.1200]
Asia	13	1624	259	0.1595 [0.1420; 0.1782]	0.1068 [0.0394; 0.2582]
North America	6	1274	161	0.1264 [0.1084; 0.1459]	0.1338 [0.0714; 0.2370]
South America	2	83	1	0.0120 [0.0003; 0.0663]	0.0203 [0.0041; 0.0949]
Oceania	2	5	0	0.0000 [0.0000; 0.5218]	0.1441 [0.0198; 0.5839]
Total	83	14,088	1445	0.1026 [0.0976; 0.1077]	0.0666 [0.0426; 0.1026]

*No. reports*: reports in this analysis included studies that reported individual arthropods (not pools of arthropods). Studies that reported arthropod data from different countries were analyzed separately based on country (e.g., if one study reported arthropods from three countries, we reported the data separately by country). *No. arthropods*: total number of arthropods tested for *B. quintana*. *No. Bq+*: number of arthropods with *B. quintana* DNA. *Bq proportion*: “No. arthropods” divided by “No. Bq+” with 95% confidence interval (CI) using the binomial "exact" calculation (one-sided 97.5% CI when 0 *B. quintana* detected). *Random-effects model proportion*: pooled proportion of arthropods with detection of *B. quintana* DNA using random-effects model, using the inverse variance method, logit transformation, and normal approximation confidence interval for individual studies and a continuity correction of 0.5 in studies with zero cell frequencies

### Arthropod genera, species, and ecotypes and associated *B. quintana* detection

Fifty-six studies tested 8839 body lice, of which 1679 harbored *B. quintana* DNA (0.1899, 95% CI [0.1818; 0.1983]) (Table 2), generating a random-effects model pooled prevalence of 0.2312 (95% CI [0.1784; 0.2843]), with high heterogeneity (*I*^2^ = 98.7%, Cochran’s *Q*, *P* < 0.0001). Forty-two studies tested 4962 head lice, of which 390 head lice from 20 studies originating from 11 different countries harbored *B. quintana* DNA (0.0786, 95% CI [0.0713; 0.0864]). This generated a random-effects model pooled prevalence for head lice of 0.0301 (95% CI [0.0221; 0.0372]), again with high heterogeneity (*I*^2^ = 90.09%, Cochran’s *Q*, *P* < 0.001). Fourteen studies tested both body and head lice, of which nine confirmed louse ecotype using PCR (Additional file 4), and five documented greater detection among head lice than body lice (Table 3) [17, 28, 30, 36, 37]. Co-infestation of body lice with head lice was documented in eight studies among a minority of hosts (Appendix 4).

**Table 2:** Arthropod type, number of studies, and *B. quintana* detection

Arthropod		No. studies	No. arthropods	No. *B. quintana* detected	*B. quintana* proportion [95% CI]
Pediculidae (lice)	*Pediculus humanus humanus* (body lice)	56	8839	1679	0.1899 [0.1818; 0.1983]
	*Pediculus humanus* *Capitis *(head lice)	42	4962	390	0.0786 [0.0713; 0.0864]
	*Pthirus pubis* (pubic lice)	2	67	2	0.0298 [0.0036; 0.1037]
	*Pedicinus obtusus* (macaque lice)	1	N/D	N/D	N/D
Cimicidae (bedbugs)	*Cimex lectularius *(cosmopolitan bedbug)	1	1	0	N/A
	*Cimex hemipterus *(tropical bedbug)	1	100	1	0.01 [0.0002; 0.0545]
Siphonaptera (fleas, multiple families)	*Pulex irritans* (human flea)	2	40	3	0.075 [0.0157; 0.2039]
	*Ctenocephalides felis* (cat flea)	4	192	14	0.0729 [0.0404; 0.1193]
	*Ctenocephalides canis* (dog flea)	1	4	0	N/A
	*Xenopsylla cheopis* (rodent flea)	2	584	9	0.0154 [0.0071; 0.0291]
Argasidae and Ixodida (ticks)^a^	Hard tick (multiple species)	2	551	0	0.0000 [0.0000; 0.0067]
	Soft tick (*Ornithodoros sawaii*)	1	74	0	0.0000 [0.0000; 0.0486]
Arachnida (mites)	*Demodex* spp.	1	72	3	0.0417 [0.0087; 0.1170]
	*Dermanyssus* spp.	1	7	7	N/A

*No. studies*: number of studies that tested arthropod type for *B. quintana*. Certain studies tested multiple types of arthropods (e.g., 17 studies tested for both body lice and head lice), which explains the discrepancy with the total number of included studies. *No. arthropods*: total number of arthropods tested for *B. quintana*. *No. B. quintana detected*: number of arthropods with *B. quintana* DNA detection. *Percentage B. quintana detected*: percentage of arthropods with *B. quintana* DNA detection and 95% confidence interval

^a^Studies with 0% or 100% of arthropods with evidence of *B. quintana* DNA were evaluated with a one-sided 97.5% confidence interval. *N/D*: no data, as *Pedicinus obtusus* lice from one study (Li et al. 2013) of 10 monkeys were separated into two pools per monkey (20 pools total) and *B. quintana* was identified in all lice pools. *N/A*: Proportions were not reported for species with fewer than 10 arthropods. Ticks were divided into hard ticks and soft ticks due to the small number of studies and the fact that the two studies of hard ticks pooled multiple species from different genera (e.g., *Ixodes*, *Dermacentor*, *Rhipicephalus*, *Hyalomma*, *Amblyomma*, *Haemaphysalis*)

**Table 3:** Studies where *B. quintana* detection in head lice exceeded that of body lice

Author	Country	Body lice Bq+ proportion [95% CI]	Head lice Bq+ proportion [95% CI]	No. hosts Bq+
Poudel 2023 [37]	Nepal	0.0667 [0.0017; 0.3195]	0.1053 [0.0130; 0.3314]	3
Perez-Tanoira 2020 [36]	Ethiopia	0.0714 [0.0018; 0.3387]	0.1875 [0.0405; 0.4565]	N/D
Bonilla 2014 [17]	USA	0.1587 [0.0788; 0.2726]	0.3750 [0.1520; 0.6457]	16
Sangare 2014 [30]	Madagascar	0.0267 [0.0032; 0.0930]	0.0455 [0.0012; 0.2284]	N/D
Cutler 2012 [28]	Ethiopia	0.0303 [0.0008; 0.1576]	0.0923 [0.0346; 0.1902]	7

All studies in the table tested both head lice and body lice. *Body lice Bq+ proportion*: proportion of body lice with *B. quintana* DNA. *Head lice Bq+ proportion*: proportion of head lice with *B. quintana* DNA. *No. hosts Bq+*: number of hosts harboring lice with *B. quintana* DNA. *N/D*: no data, as host information not reported

Overall, the presence of *B. quintana* DNA was greater among body lice than head lice (*χ*^2^ = 308.3, *df* = 1, two-tailed *P* < 0.0001). However, nine studies that exclusively tested head lice detected *B. quintana* DNA (Table 4). All nine of these studies collected arthropods from low-income contexts: seven were from low- and middle-income countries (LMICs), and of the two from high-income countries, one was from rural Georgia, USA (an area known for poverty), and one from an individual experiencing homelessness in France (Table 4) [18, 38]. Head lice collected from schoolchildren in high-income countries such as France, Portugal, and Australia had no evidence of *B. quintana* DNA (Additional file 5).

**Table 4:** Studies that exclusively tested head lice and reported *B. quintana* detection

Author	Country	No. head lice	No. Bq+	Bq+ proportion [95% CI]	No. hosts	No. hosts Bq+	Host age
Hammoud [16]^a^	Senegal	161	93	0.5776[0.4974; 0.6550]	N/D	5	Adults and children
Dzul-Rosado 2022 [33]^b^	Mexico	28	2	0.0714 [0.0088; 0.2350]	28	2	Children
Eremeeva 2019 [47]^b^	Madagascar	159	20	0.1258 [0.0786; 0.1876]	39	N/D	Adults and children
Ulutasdemir 2018 [32]^a^	Turkey	26	3	0.1154 [0.0245; 0.3015]	N/D	N/D	N/D
Eremeeva-1 2017 [38]^a^	USA	178	21	0.1180 [0.0745; 0.1747]	N/D	N/D	N/D
Amanzougaghene 2017 [48]^a^	Mali	600	3	0.0050 [0.0010; 0.0145]	117	2	Adults and children
Diatta 2014 [42]^a^	Senegal	148	2	0.0135[0.0016; 0.0480]	40	2	Adults and children
Boutellis 2012 [53]^a^	Senegal	274	19	0.0693[0.0423; 0.1062]	100	7	Adults and children
Angelakis 2011^a^ [18]^b^	France	N/D	N/D	N/D	1	1	Adults

*No. head lice*: number of head lice in the study. *No. Bq+*: number of head lice testing positive for *B. quintana* DNA. *Bq+ proportion*: proportion of head lice that tested positive for *B. quintana* DNA. *No. hosts*: total number of hosts in the study. *No. hosts Bq+*: number of hosts with head lice that tested positive for *B. quintana* DNA. ^a^ Arthropod species/ecotype identified using PCR. ^b^ Arthropod species/ecotype identified using visualization of lice in head hair or collected from hair combs. *N/D*: no data available

^a^In Angelakis et al., a single individual was reported to have *B. quintana* DNA-positive head lice, but this study did not report the number of lice tested

Regarding non-*Pediculus* arthropods, one of the two studies that tested 67 pubic lice (*Pthirus pubis*) reported the presence of *B. quintana* DNA among two lice from one individual. One study tested macaque lice (*Pedicinus obtusus*) from 10 non-human primates; arthropods were separated into two pools per monkey, and *B. quintana* was identified in all lice pools [39]. Two studies tested bedbugs (Cimidae); *B. quintana* DNA was detected in one arthropod in one instance [40]. Nine studies tested 820 fleas (Siphonaptera) of various species. While *B. quintana* DNA was not detected in dog fleas, three human fleas (*Pulex irritans*), 14 cat fleas (*Ctenocephalides felis*), and nine rodent fleas (*Xenopsylla cheopis*) tested positive for *B. quintana* using molecular methods, with associated proportions of 0.075 (95% CI [0.0157; 0.2039]), 0.0729 (95% CI [0.0404; 0.1193]), and 0.0154 (95% CI [0.0071; 0.0291]), respectively. Three studies tested ticks, with two testing hard ticks (multiple species) and one testing soft ticks (*Orthodoros sawaii*); none were associated with the presence of *B. quintana* DNA. Two studies tested mites, one testing *Demodex* species and the other testing *Dermanyssus* species, with *B. quintana* detected molecularly in three and seven mites, respectively [15, 41].

### *Bartonella quintana*-related disease among hosts

Host clinical disease and the presence of *B. quintana* DNA in blood samples were reported in seven studies. Clinical disease was most commonly described as fever [3]. All hosts with *B. quintana* DNA in blood samples were human. Five studies described infestations with *B. quintana* DNA-positive body lice, two studies described infestation with *B. quintana* DNA-positive head lice without body lice co-infestation, and one described infestation with *B. quintana* DNA-positive *Dermanyssus* mites [15, 16, 42–44].

### Co-infection with other pathogens

Among the 31 studies that tested arthropods for pathogens other than *B. quintana*, various *Acinetobacter* species were most commonly identified in 18 studies. Despite their shared transmission with body lice, *R. prowazekii* and *B. recurrentis* were rarely detected in arthropods. *Rickettsia prowazekii* was identified in only three studies testing arthropods from refugees in Turkey and Burundi and homeless populations in Colombia [29, 32, 45]. *Borrelia recurrentis* was identified in only one study of head lice from the Republic of the Congo [46].

### Quality assessment

Quality assessment using the modified JBI critical appraisal checklist for cross-sectional studies revealed that 62 publications had sufficient information to be included in the full-text analysis (Additional file 6) [27]. Thirteen publications were excluded predominantly due to insufficient diagnostic information.

## Discussion

The detection of *B. quintana* DNA among arthropods in 37 countries across most continents suggests that *B. quintana* is a vector-borne disease with a global distribution. The elevated number of *B. quintana* DNA-positive arthropods from certain African and Asian countries suggests a considerable burden in LMICs [30, 37]. While *B. quintana* is often described as a rare pathogen, our description of *B. quintana* among 1445 arthropods from 293 hosts indicates that *B. quintana* may be more common than previously believed. However, additional data are needed to fully elucidate the epidemiology of *B. quintana*. This review highlights how entomologic studies may provide a non-invasive method of facilitating *B. quintana* surveillance among key populations, such as those with a history of homelessness, incarceration, or forced displacement/immigration from certain LMICs. Although data were reported according to country, *B. quintana* detection among arthropods is associated with poverty: schoolchildren with head lice from high-income environments are unlikely to harbor *B. quintana* and wealthy adults from LMICs are unlikely to have pediculosis*.* Entomologic surveillance of *B. quintana* would have the highest yield when applied only to specific key populations. Certain countries, such as Brazil, Colombia, Madagascar, Mali, Nigeria, and Peru, reported *B. quintana* DNA-positive arthropods but have no published cases of *B. quintana* endocarditis, indicating that arthropod studies may identify a hidden burden of disease in LMICs [13, 31, 43, 45, 47, 48]. Many countries had a limited number of studies or no published data, suggesting that arthropod testing for *B. quintana* is an underutilized surveillance tool [41, 49].

While this review summarized *B. quintana* detection among many different arthropod species, not all of these arthropods meet criteria to be considered vectors for *B. quintana*. To be considered a vector, transmission between hosts needs to be demonstrated [50]. Detection of *B. quintana* DNA is insufficient to deem an arthropod a vector, as the arthropod may have acquired *B. quintana* through a blood meal but remain incapable of transmitting the bacterium to a new host. This “dead-end” acquisition of *B. quintana* may be enhanced by the known chronicity of *B. quintana* bacteremia [1, 3]. Transmission of *B. quintana* from body lice to human hosts involves the inoculation of infected body louse feces into skin abrasions and mucous membranes [1, 51]. Arthropods that do not defecate at the time of feeding and do not live on or close to the host are unlikely to transmit *B. quintana*. These arthropod species may be more likely to demonstrate dead-end DNA acquisition without vector competence. Furthermore, *B. quintana* transmission requires bacterial multiplication in the arthropod gut. Arthropods that consume and digest a blood meal with *B. quintana* may have fragments of *B. quintana* identified by PCR without any live bacteria, precluding transmission to the next host*.*

While WWI xenodiagnostic studies have long established *B. quintana* transmission via body lice, transmission via head lice has been demonstrated more recently and less frequently [16]. Only two studies describe the presence of *B. quintana* DNA in human blood samples linked to head lice infestation [16, 42]. In one recent study describing a Senegalese outbreak, *B. quintana* transmission via head lice (without concomitant body lice infestation) was supported by genomic analyses, with 99.98% similarity between *B. quintana* acquired from blood and head lice samples [16]. For non-*Pediculus* arthropods, only a single study of macaque lice (*P. obtusus*) and of *Dermanyssus* pigeon mites have shown a link to host infection [15, 39]. However, these findings have not been replicated, and additional data are needed to explore whether arthropods beyond *P. humanus* may transmit *B. quintana* [15, 39] While bedbugs (Cimidae) have demonstrated vector competence for *B. quintana* in vitro, no studies have demonstrated *B. quintana* transmission in vivo [23].

Despite the infrequency of proven transmission via head lice, the presence of *B. quintana* DNA among head lice is not rare, but appears to depend on the socioeconomic context [17, 37, 38]. While all studies of head lice collected from schoolchildren in high-resource countries are *B. quintana*-negative [29], *B. quintana* DNA may be detected among head lice from adults and children in low-resource settings [28, 30, 33, 37]. The reason that head lice among schoolchildren in high-income contexts test negative for *B. quintana* is unknown but may relate less to the absence of vector competence and more to epidemiology: if not belonging to the key populations mentioned above, schoolchildren in high-income contexts are unlikely to be in close contact with *B. quintana*-bacteremic individuals. In high-income countries, *B. quintana* among head lice is limited to low-resource contexts such as urban homelessness or rural impoverishment [17, 18, 47]. In the USA, *B. quintana* DNA has been detected among head lice collected from homeless populations in San Francisco and housed populations in Georgia, an area known for rural poverty [17, 47].

The presence of *B. quintana* in head lice may not require co-infestation of body lice with head lice in a single individual. Among the 13 studies that analyzed both body and head lice, co-infestation of body and head lice was documented in eight studies with rates as low as 3% among a homeless population in the USA [17]. The presence of *B. quintana* in head lice from different environments suggests that future introductions into high-income populations of school-age children may be possible should these children share close contact with individuals with ongoing *B. quintana* bacteremia and pediculosis.

While *R. prowazekii* and *B. recurrentis* are frequently described as louse-borne, very few studies have detected DNA of these pathogens, indicating that *B. quintana* may be the most common louse-borne disease [29, 32, 46, 45]. Certain high-income jurisdictions have described imported cases of *B. recurrentis* but have not reported cases of *B. quintana* [13, 52]*.* This relative predominance of *B. recurrentis* may reflect diagnostic bias: *B. recurrentis* is visible on Giemsa stains performed for malaria while *B. quintana* is not [52]. The preponderance of *B. quintana* in arthropods implies that any cases of *B. recurrentis* and *R. prowazekii* should also be tested for *B. quintana* infection.

This systematic review is subject to several limitations. All included articles necessitated confirmation of *B. quintana* to species level, which inherently created a bias towards recent studies that used molecular diagnostics. Significant heterogeneity was found between studies. The data may have been influenced by publication bias and other forms of bias in the original studies. It is possible that some head lice studies included patients with undocumented concomitant or previous body lice infestation. Heterogeneity in study methodology prevented the inclusion of a minority of articles in the random-effects models of prevalence. Statistical analyses were applied post hoc, increasing the risk of false discovery.

## Conclusions

This systematic review reveals that *B. quintana* is a louse-borne disease with a global distribution and a disproportionate burden in low-resource settings. While less commonly infected than body lice, a limited number of studies suggest that head lice may also act as vectors for *B. quintana* in specific low-resource contexts. Prospective studies that simultaneously test arthropods for *B. quintana* carriage and human hosts for *B. quintana* infection/disease are needed to elucidate transmission rates and proportions of severe illness, such as endocarditis. These studies are necessary to determine whether individuals with *B. quintana* DNA-positive ectoparasites should be treated for *B. quintana* infection, especially in areas where molecular testing and echocardiography are difficult to obtain. We encourage public health laboratories to include *B. quintana* testing of body lice (and head lice originating from low-resource contexts) as a surveillance modality to improve our understanding of the epidemiology of this neglected disease.

### Supplementary Information

Supplementary Material 1.

Supplementary Material 2.

Supplementary Material 3.

Supplementary Material 4.

Supplementary Material 5.

Supplementary Material 6.

## Data Availability

Data is available in attached appendices.

## Footnotes

1 We are aware that Corsica is politically part of France; however, we have treated it as a separate jurisdiction due to its geographical isolation from mainland France and the different results of its arthropod studies.
