# Supplementary material for: Bartonella quintana detection among arthropods and their hosts: a systematic review and meta-analysis
Source: Parasit Vectors. 2024 Aug 2;17:328. doi: 10.1186/s13071-024-06413-3 (PMC11295871; doi:10.1186/s13071-024-06413-3)
Supplement: Supplementary file 1 — Supplementary Material 1. [file 13071_2024_6413_MOESM1_ESM.docx]

checklist for analytical cross sectional studies

Critical Appraisal tools for use in JBI Systematic Reviews

Introduction

JBI is an JBI is an international research organisation based in the Faculty of Health and Medical Sciences at the University of Adelaide, South Australia. JBI develops and delivers unique evidence-based information, software, education and training designed to improve healthcare practice and health outcomes. With over 70 Collaborating Entities, servicing over 90 countries, JBI is a recognised global leader in evidence-based healthcare.

## JBI Systematic Reviews

The core of evidence synthesis is the systematic review of literature of a particular intervention, condition or issue. The systematic review is essentially an analysis of the available literature (that is, evidence) and a judgment of the effectiveness or otherwise of a practice, involving a series of complex steps. JBI takes a particular view on what counts as evidence and the methods utilised to synthesise those different types of evidence. In line with this broader view of evidence, JBI has developed theories, methodologies and rigorous processes for the critical appraisal and synthesis of these diverse forms of evidence in order to aid in clinical decision-making in healthcare. There now exists JBI guidance for conducting reviews of effectiveness research, qualitative research, prevalence/incidence, etiology/risk, economic evaluations, text/opinion, diagnostic test accuracy, mixed-methods, umbrella reviews and scoping reviews. Further information regarding JBI systematic reviews can be found in the [JBI Evidence Synthesis Manual](https://jbi-global-wiki.refined.site/space/MANUAL).

## JBI Critical Appraisal Tools

All systematic reviews incorporate a process of critique or appraisal of the research evidence. The purpose of this appraisal is to assess the methodological quality of a study and to determine the extent to which a study has addressed the possibility of bias in its design, conduct and analysis. All papers selected for inclusion in the systematic review (that is – those that meet the inclusion criteria described in the protocol) need to be subjected to rigorous appraisal by two critical appraisers. The results of this appraisal can then be used to inform synthesis and interpretation of the results of the study. JBI Critical appraisal tools have been developed by the JBI and collaborators and approved by the JBI Scientific Committee following extensive peer review. Although designed for use in systematic reviews, JBI critical appraisal tools can also be used when creating Critically Appraised Topics (CAT), in journal clubs and as an educational tool.

Modified JBI Critical Appraisal Checklist for
analytical cross sectional studies

Reviewer ______________________________________ Date_______________________________

Author_______________________________________ Year_________ Record Number_________

|  | Yes | No | Unclear | Not applicable |
| --- | --- | --- | --- | --- |
| 1. Were the criteria for inclusion in the sample clearly defined, with arthropods collected in the field (not laboratory raised)? | □ | □ | □ | □ |
| 1. Were arthropods and the setting described in detail (eg: arthropods diagnosed to genus-level or species-level)? | □ | □ | □ | □ |
| 1. Was *Bartonella quintana* tested to species-level using objective, standard criteria used for measurement of the condition? | □ | □ | □ | □ |
| 1. Was the host population and host infection/ disease described in detail? | □ | □ | □ | □ |
| 1. Was appropriate statistical analysis used? | □ | □ | □ | □ |

Overall appraisal: Include □ Exclude □ Seek further info □

Comments (Including reason for exclusion)

________________________________________________________________________________________________________________________________________________________________________________________________________________________________________________________________________________________________

Explanation of analytical cross sectional studies critical appraisal (Modified)

*How to cite:* Moola S, Munn Z, Tufanaru C, Aromataris E, Sears K, Sfetcu R, Currie M, Qureshi R, Mattis P, Lisy K, Mu P-F. Chapter 7: Systematic reviews of etiology and risk . In: Aromataris E, Munn Z (Editors). JBI Manual for Evidence Synthesis. JBI, 2020. Available from <https://synthesismanual.jbi.global>

**Analytical cross sectional studies Critical Appraisal Tool**

Answers: Yes, No, Unclear or Not/Applicable

## 1. Were the criteria for inclusion in the sample clearly defined, with arthropods collected in the field (not laboratory raised)?

The authors should provide clear inclusion and exclusion criteria that they developed prior to arthropod sampling. Arthropods must be collected in the field for inclusion. No in vitro/ laboratory-raised arthropods will be included.

## 2. Were arthropods and the setting described in detail (eg: arthropods diagnosed to genus-level or species-level)?

The study arthropods should be described in sufficient taxonomic detail (at least to genus). The authors should provide a clear description of the population from which the arthropods were selected including demographics, location, and time period.

## 3. Was *Bartonella quintana* tested to species-level using objective, standard criteria used for measurement of the condition?

The study should clearly describe the method of testing *Bartonella quintana* to species-level. Articles that test to genus-level will be deemed inadequate and will be excluded.

## 4. Was the host population and host infection/ disease described in detail?

It is useful to determine if patients were included in the study based on either a specified diagnosis or definition. While not essential for inclusion in this systematic review, articles that include data on host population and associated *Bartonella quintana* related infection and disease will be deemed to have higher quality.

## 8. Was appropriate statistical analysis used?

As with any consideration of statistical analysis, consideration should be given to whether there was a more appropriate alternate statistical method that could have been used. The methods section should be detailed enough for reviewers to identify which analytical techniques were used (in particular, regression or stratification) and how specific confounders were measured.

For studies utilizing regression analysis, it is useful to identify if the study identified which variables were included and how they related to the outcome. If stratification was the analytical approach used, were the strata of analysis defined by the specified variables? Additionally, it is also important to assess the appropriateness of the analytical strategy in terms of the assumptions associated with the approach as differing methods of analysis are based on differing assumptions about the data and how it will respond.
