# Supplementary material for: Bartonella quintana detection among arthropods and their hosts: a systematic review and meta-analysis
Source: Parasit Vectors. 2024 Aug 2;17:328. doi: 10.1186/s13071-024-06413-3 (PMC11295871; doi:10.1186/s13071-024-06413-3)
Supplement: Supplementary file 3 — Supplementary Material 3. [file 13071_2024_6413_MOESM3_ESM.docx]

**Random effects model: all arthropods global**

Proportion 95%-CI %W(random)

Tufa 2023 0.1304 [0.0427; 0.3355] 1.6,

Kamani 2023 0.0489 [0.0273; 0.0861] 1.7

Jumpertz 2023 0.0000 [0.0001; 0.0188] 1.1

Hammoud 2023 0.5776 [0.4974; 0.6550] 1.6

Poudel 2023 0.0151 [0.0049; 0.0457] 1.6

Dzul-Rosado 2022 0.0714 [0.0179; 0.2448] 1.5

Sasaki 2021 0.6897 [0.5030; 0.8299] 1.7

Hammoud 2021 0.0000 [0.0002; 0.0491] 1.1

Pietri 2020 0.0000 [0.0095; 0.8064] 1.0

Majid 2020 0.0000 [0.0031; 0.4749] 1.1

Bounmbanda-koyo 2020 0.0000 [0.0000; 0.0114] 1.1

Amanzougaghene 2020 0.0493 [0.0267; 0.0891] 1.7

Ly 2020 0.0119 [0.0053; 0.0261] 1.7

Perez-Tanoira 2020 0.0331 [0.0125; 0.0848] 1.6

Theonest 2019 0.0000 [0.0001; 0.0153] 1.1

Ly 2019 0.0102 [0.0046; 0.0225] 1.7

Huang 2019 0.0000 [0.0005; 0.1179] 1.1

De Liberato 2019 0.0000 [0.0011; 0.2364] 1.1

Bounmbanda-koyo 2019 0.0000 [0.0002; 0.0423] 1.1

Eremeeva 2019 0.1258 [0.0826; 0.1869] 1.7

Ulutasdemir 2018 0.1154 [0.0377; 0.3029] 1.6

Louni 2018 0.0000 [0.0004; 0.0890] 1.1

Louni 2018 0.1336 [0.1070; 0.1655] 1.8

Candy 2018 0.0154 [0.0058; 0.0404] 1.6

Perez-Tanoira 2017 0.0756 [0.0398; 0.1390] 1.7

Mana 2017 0.0000 [0.0005; 0.1055] 1.1

Eremeeva 2017 0.3552 [0.3104; 0.4027] 1.8

Amanzougaghene 2017 0.0050 [0.0016; 0.0154] 1.6

Amanzougaghene 2016 0.0000 [0.0000; 0.0125] 1.1

Drali 2015 0.3103 [0.2461; 0.3829] 1.8

Sangare 2014 0.2288 [0.2043; 0.2554] 1.8

Murillo 2014 0.0417 [0.0135; 0.1214] 1.6

Drali 2014 0.1881 [0.1649; 0.2136] 1.8

Sangare 2015 0.0000 [0.0003; 0.0801] 1.1

Bonilla 2014 0.2025 [0.1279; 0.3054] 1.7

Diatta 2014 0.0135 [0.0034; 0.0524] 1.5

Angelakis 2013 0.0100 [0.0014; 0.0675] 1.3

Boutellis 2012 0.0693 [0.0447; 0.1061] 1.7

Melter 2012 1.0000 [0.4614; 0.9962] 1.1

Cutler 2012 0.0686 [0.0331; 0.1370] 1.7

Bouvresse 2011 0.0000 [0.0001; 0.0270] 1.1

Angelakis 2011 0.1367 [0.1131; 0.1643] 1.8

Alcantara 2009 0.2833 [0.2346; 0.3376] 1.8

Bonilla 2009 0.0562 [0.0413; 0.0760] 1.8

Sasaki2 2006 0.0840 [0.0625; 0.1121] 1.8

Marié 2006 0.1169 [0.0619; 0.2096] 1.7

Rolain 2005 0.7500 [0.2378; 0.9665] 1.3

Rolain 2003 0.1728 [0.1051; 0.2710] 1.7

Fournier-1 2002 0.0988 [0.0707; 0.1363] 1.7

Fournier-2 2002 0.3600 [0.1991; 0.5600] 1.7

Fournier-3 2002 0.0000 [0.0073; 0.7344] 1.0

Fournier-4 2002 0.0000 [0.0009; 0.1959] 1.1

Fournier-5 2002 0.2105 [0.1089; 0.3678] 1.7

Fournier-6 2002 0.9009 [0.8299; 0.9443] 1.7

Fournier-7 2002 0.9394 [0.7877; 0.9848] 1.5

Fournier-8 2002 0.0229 [0.0103; 0.0500] 1.7

Fournier-9 2002 0.0000 [0.0095; 0.8064] 1.0

Fournier-10 2002 0.0000 [0.0028; 0.4483] 1.1

Fournier-11 2002 0.0000 [0.0002; 0.0530] 1.1

La Scola 2001 0.2547 [0.1933; 0.3275] 1.7

Rydkina 1999 0.1228 [0.0886; 0.1677] 1.7

Roux-1 1999 0.0000 [0.0038; 0.5386] 1.1

Roux-2 1999 0.1667 [0.0420; 0.4772] 1.5

Roux-3 1999 0.0000 [0.0028; 0.4483] 1.1

Roux-4 1999 0.0952 [0.0434; 0.1962] 1.7

Roux-5 1999 0.1429 [0.0848; 0.2307] 1.7

Roux-6 1999 0.0400 [0.0130; 0.1168] 1.6

Roux-7 1999 0.1231 [0.0889; 0.1682] 1.7

Roux-8 1999 0.0137 [0.0019; 0.0909] 1.3

Number of studies: k = 68

Number of observations: o = 14564

Number of events: e = 1454.9

proportion 95%-CI

Random effects model 0.0666 [0.0426; 0.1026]

Quantifying heterogeneity:

tau^2 = 3.2834 [2.3883; 5.6702]; tau = 1.8120 [1.5454; 2.3812]

I^2 = 93.3% [92.1%; 94.3%]; H = 3.86 [3.57; 4.19]

Test of heterogeneity:

Q d.f. p-value

1000.84 67 < 0.0001

Details on meta-analytical method:

- Inverse variance method

- Restricted maximum-likelihood estimator for tau^2

- Q-Profile method for confidence interval of tau^2 and tau

- Logit transformation

- Normal approximation confidence interval for individual studies

- Continuity correction of 0.5 in studies with zero cell frequencies

**Regional pooled prevalence random effects model:**

**Africa**

proportion 95%-CI %W(random)

Roux-1 0.0000 [0.0000; 0.4096] 1.9

Roux-2 0.1667 [0.0209; 0.4841] 2.4

Roux-3 0.0000 [0.0000; 0.3085] 1.9

Roux-4 0.0952 [0.0358; 0.1959] 2.6

Roux-5 0.1429 [0.0783; 0.2319] 2.7

Fournier-14 0.0000 [0.0000; 0.1853] 1.9

Fournier-15 0.0000 [0.0000; 0.1684] 1.9

Fournier-3 0.0000 [0.0000; 0.7076] 1.9

Fournier-4 0.0000 [0.0000; 0.1058] 1.9

Fournier-5 0.2105 [0.0955; 0.3732] 2.7

Fournier-6 0.9009 [0.8296; 0.9495] 2.7

Fournier-7 0.9394 [0.7977; 0.9926] 2.5

Fournier-8 0.0229 [0.0084; 0.0492] 2.6

Rolain 0.7500 [0.1941; 0.9937] 2.1

Angelakis 0.1367 [0.1120; 0.1645] 2.7

Boutellis 0.0693 [0.0423; 0.1062] 2.7

Cutler 0.0686 [0.0280; 0.1363] 2.7

Angelakis 0.0100 [0.0003; 0.0545] 2.3

Diatta 0.0135 [0.0016; 0.0480] 2.5

Sangare-1 0.0000 [0.0000; 0.1157] 1.9

Sangare-2 0.0309 [0.0064; 0.0877] 2.6

Sangare-3 0.0000 [0.0000; 0.1234] 1.9

Sangare-4 0.0000 [0.0000; 0.0393] 1.9

Sangare-5 0.7297 [0.5588; 0.8621] 2.7

Sangare-6 0.8976 [0.8411; 0.9392] 2.7

Sangare-7 0.0500 [0.0013; 0.2487] 2.2

Sangare-8 0.2963 [0.2322; 0.3669] 2.7

Drali 0.3103 [0.2425; 0.3848] 2.7

Sangare 0.0000 [0.0000; 0.0393] 1.9

Amanzougaghene 0.0000 [0.0000; 0.0058] 1.9

Amanzougaghene 0.0050 [0.0010; 0.0145] 2.6

Mana 0.0000 [0.0000; 0.0528] 1.9

Perez-Tanoira 0.0756 [0.0352; 0.1387] 2.7

Louni 0.0000 [0.0000; 0.0440] 1.9

Louni 0.1336 [0.1056; 0.1657] 2.7

Bounmbanda-koyo 0.0000 [0.0000; 0.0202] 1.9

Eremeeva 0.1258 [0.0786; 0.1876] 2.7

Theonest 0.0000 [0.0000; 0.0072] 1.9

Bounmbanda-koyo 0.0000 [0.0000; 0.0053] 1.9

Perez-Tanoira 0.0331 [0.0091; 0.0825] 2.6

Hammoud 2023 0.5776 [0.4974; 0.6550] 1.6

Hammoud 0.0000 [0.0000; 0.0235] 1.9

Kamani 0.0489 [0.0247; 0.0858] 2.7

Tufa 0.1304 [0.0278; 0.3359] 2.5

Number of studies: k = 43

Number of observations: o = 7040

Number of events: e = 703

proportion 95%-CI

**Random effects model 0.0615 [0.0311; 0.1180]**

Quantifying heterogeneity:

tau^2 = 4.8439 [3.0241; 8.3405]; tau = 2.2009 [1.7390; 2.8880]

I^2 = 94.6% [93.5%; 95.5%]; H = 4.30 [3.92; 4.73]

Test of heterogeneity:

Q d.f. p-value

777.88 42 < 0.0001

Details on meta-analytical method:

- Inverse variance method

- Restricted maximum-likelihood estimator for tau^2

- Q-Profile method for confidence interval of tau^2 and tau

- Logit transformation

- Clopper-Pearson confidence interval for individual studies

- Continuity correction of 0.5 in studies with zero cell frequencies

**Asia**

proportion 95%-CI %W(random)

Roux-7 0.1231 [0.0889; 0.1682] 9.2

Rydkina 0.1228 [0.0886; 0.1677] 9.2

Fournier-13 0.0000 [0.0028; 0.4483] 5.6

Fournier-16 0.0000 [0.0013; 0.2594] 5.7

Fournier-17 0.0000 [0.0010; 0.2172] 5.7

Marié 0.1169 [0.0619; 0.2096] 9.0

Sasaki 0.0840 [0.0625; 0.1121] 9.3

Eremeeva-2 0.8478 [0.7778; 0.8986] 9.2

Ulutasdemir 0.1154 [0.0377; 0.3029] 8.4

Huang 0.0000 [0.0005; 0.1179] 5.7

Majid 0.0000 [0.0031; 0.4749] 5.6

Sasaki 0.6897 [0.5030; 0.8299] 8.9

Poudel 0.0151 [0.0049; 0.0457] 8.4

Number of studies: k = 13

Number of observations: o = 1624

Number of events: e = 258.9

proportion 95%-CI

Random effects model 0.1068 [0.0394; 0.2582]

Quantifying heterogeneity:

tau^2 = 3.1834 [1.3327; 9.0028]; tau = 1.7842 [1.1544; 3.0005]

I^2 = 96.0% [94.4%; 97.1%]; H = 4.97 [4.23; 5.84]

Test of heterogeneity:

Q d.f. p-value

296.49 12 < 0.0001

Details on meta-analytical method:

- Inverse variance method

- Restricted maximum-likelihood estimator for tau^2

- Q-Profile method for confidence interval of tau^2 and tau

- Logit transformation

- Normal approximation confidence interval for individual studies

- Continuity correction of 0.5 in studies with zero cell frequencies

**Europe**

proportion 95%-CI %W(random)

Roux-6 0.0400 [0.0083; 0.1125] 6.4

La Scola 0.2547 [0.1894; 0.3292] 7.0

Fournier-1 0.0988 [0.0685; 0.1366] 7.0

Fournier-11 0.0000 [0.0000; 0.1684] 4.2

Fournier-12 0.0000 [0.0000; 0.1684] 4.2

Fournier-2 0.3600 [0.1797; 0.5748] 6.7

Rolain 0.1728 [0.0978; 0.2730] 6.9

Bouvresse 0.0000 [0.0000; 0.0127] 4.3

Melter 1.0000 [0.5904; 1.0000] 4.2

Drali 0.1881 [0.1642; 0.2138] 7.1

Murillo 0.0417 [0.0087; 0.1170] 6.4

Candy 0.0154 [0.0042; 0.0391] 6.6

De Liberato 0.0000 [0.0000; 0.1323] 4.2

Ly 0.0102 [0.0038; 0.0221] 6.7

Amanzougaghene 0.0493 [0.0239; 0.0887] 6.9

Ly 0.0119 [0.0044; 0.0256] 6.7

Jumpertz 0.0000 [0.0000; 0.0088] 4.3

Number of studies: k = 17

Number of observations: o = 4062

Number of events: e = 321

proportion 95%-CI

Random effects model 0.0522 [0.0218; 0.1200]

Quantifying heterogeneity:

tau^2 = 3.0005 [1.5264; 10.1076]; tau = 1.7322 [1.2355; 3.1792]

I^2 = 92.6% [89.7%; 94.7%]; H = 3.68 [3.12; 4.35]

Test of heterogeneity:

Q d.f. p-value

217.00 16 < 0.0001

Details on meta-analytical method:

- Inverse variance method

- Restricted maximum-likelihood estimator for tau^2

- Q-Profile method for confidence interval of tau^2 and tau

- Logit transformation

- Clopper-Pearson confidence interval for individual studies

- Continuity correction of 0.5 in studies with zero cell frequencies

**North America**

proportion 95%-CI %W(random)

Alcantara 0.2833 [0.2324; 0.3386] 22.0

Bonilla 0.0562 [0.0403; 0.0760] 21.6

Bonilla 0.2025 [0.1204; 0.3080] 19.9

Dzul-Rosado 0.0714 [0.0088; 0.2350] 11.6

Eremeeva-1 0.1180 [0.0745; 0.1747] 20.7

Pietri 0.0000 [0.0000; 0.8419] 4.3

Number of studies: k = 6

Number of observations: o = 1274

Number of events: e = 161

proportion 95%-CI

Random effects model 0.1338 [0.0714; 0.2370]

Quantifying heterogeneity:

tau^2 = 0.5598 [0.1425; 2.9909]; tau = 0.7482 [0.3774; 1.7294]

I^2 = 94.2% [89.9%; 96.7%]; H = 4.15 [3.15; 5.47]

Test of heterogeneity:

Q d.f. p-value

86.25 5 < 0.0001

Details on meta-analytical method:

- Inverse variance method

- Restricted maximum-likelihood estimator for tau^2

- Q-Profile method for confidence interval of tau^2 and tau

- Logit transformation

- Clopper-Pearson confidence interval for individual studies

- Continuity correction of 0.5 in studies with zero cell frequencies

**Oceania**

proportion 95%-CI %W(random)

Fournier-18 0.0000 [0.0000; 0.7076] 51.2

Fournier-9 0.0000 [0.0000; 0.8419] 48.8

Number of studies: k = 2

Number of observations: o = 5

Number of events: e = 0

proportion 95%-CI

Random effects model 0.1441 [0.0198; 0.5839]

Quantifying heterogeneity:

tau^2 = 0; tau = 0; I^2 = 0.0%; H = 1.00

Test of heterogeneity:

Q d.f. p-value

0.02 1 0.8765

Details on meta-analytical method:

- Inverse variance method

- Restricted maximum-likelihood estimator for tau^2

- Logit transformation

- Clopper-Pearson confidence interval for individual studies

- Continuity correction of 0.5 in studies with zero cell frequencies

**South America**

**> summary (Regional_South_America)**

proportion 95%-CI % W(random)

Fournier-10 0.0000 [0.0000; 0.3085] 32.6

Roux-8 0.0137 [0.0003; 0.0740] 67.4

Number of studies: k = 2

Number of observations: o = 83

Number of events: e = 1

proportion 95%-CI

Random effects model 0.0203 [0.0041; 0.0949]

Quantifying heterogeneity:

tau^2 = 0; tau = 0; I^2 = 0.0%; H = 1.00

Test of heterogeneity:

Q d.f. p-value

0.49 1 0.4847

Details on meta-analytical method:

- Inverse variance method

- Restricted maximum-likelihood estimator for tau^2

- Logit transformation

- Clopper-Pearson confidence interval for individual studies

- Continuity correction of 0.5 in studies with zero cell frequencies
